# Distinct classes and subclasses of antibodies to hemolysin co-regulated protein 1 and O-polysaccharide and correlation with clinical characteristics of melioidosis patients

**DOI:** 10.1038/s41598-019-48828-4

**Published:** 2019-09-27

**Authors:** Apinya Pumpuang, Rungnapa Phunpang, Peeraya Ekchariyawat, Adul Dulsuk, Siriorn Loupha, Kochnipa Kwawong, Yaowaree Charoensawat, Ekkachai Thiansukhon, Nicholas P. J. Day, Mary N. Burtnick, Paul J. Brett, T. Eoin West, Narisara Chantratita

**Affiliations:** 1 Department of Microbiology and Immunology, Faculty of Tropical Medicine, Mahidol University, Bangkok, Thailand; 2 Department of Clinical Pathology, Faculty of Medicine, Vajira Hospital, Navamindradhiraj University, Bangkok, Thailand; 3 Mahidol-Oxford Tropical Medicine Research Unit, Faculty of Tropical Medicine, Mahidol University, Bangkok, Thailand; 4 Department of Microbiology, Faculty of Public health, Mahidol University, Bangkok, Thailand; 5 Department of Medicine, Udon Thani Hospital, Udon Thani, Thailand; 6 Center for Tropical Medicine and Global Health, University of Oxford, Oxford, United Kingdom; 7 Department of Microbiology and Immunology, University of Nevada, Reno School of Medicine, Reno, Nevada USA; 8 Division of Pulmonary and Critical Care Medicine, Harborview Medical Center, and International Respiratory and Severe Illness Center, University of Washington, Seattle, WA USA

**Keywords:** Bacterial infection, Diagnostic markers

## Abstract

Melioidosis is a tropical infectious disease caused by *Burkholderia pseudomallei* that results in high mortality. Hemolysin co-regulated protein 1 (Hcp1) and O-polysaccharide (OPS) are vaccine candidates and potential diagnostic antigens. The correlation of classes/subclasses of antibodies against these antigens with clinical characteristics of melioidosis patients is unknown. Antibodies in plasma samples from melioidosis patients and healthy donors were quantified by ELISA and compared with clinical features. In melioidosis patients, Hcp1 induced high IgG levels. OPS induced high IgG and IgA levels. The area under receiver operating characteristic curve (AUROCC) to discriminate melioidosis cases from healthy donors was highest for anti-Hcp1 IgG (0.92) compared to anti-Hcp1 IgA or IgM. In contrast, AUROCC for anti-OPS for IgG (0.91) and IgA (0.92) were comparable. Anti-Hcp1 IgG1 and anti-OPS IgG2 had the greatest AUROCCs (0.87 and 0.95, respectively) compared to other IgG subclasses for each antigen. Survivors had significantly higher anti-Hcp1 IgG3 levels than non-survivors. Male melioidosis patients with diabetes had higher anti-OPS IgA levels than males without diabetes. Thus, diverse and specific antibody responses are associated with distinct clinical characteristics in melioidosis, confirming the diagnostic utility of these responses and providing new insights into immune mechanisms.

## Introduction

Melioidosis is an infectious disease caused by *Burkholderia pseudomallei*, an environmental Gram-negative bacterium and CDC Tier 1 select agent. The disease is highly endemic in tropical regions particularly northeast Thailand and northern Australia^1,2^. The burden of melioidosis on public health is high with an estimated 165,000 cases and 89,000 deaths annually^3^. Clinical manifestations of melioidosis range from mild to severe sepsis, and can be acute or chronic in nature. The majority of patients present with bacteremia^4^. Melioidosis is associated with several host risk factors with diabetes mellitus (DM) being one of the most important as it is reported in >50% of patients^5,6^. In northeast Thailand, melioidosis causes an overall mortality rate of ≥40% and this can reach as high as 90% in severe sepsis patients^4^. Diagnosis is based on bacterial culture, which can take several days, and there is currently no vaccine available.

Effective control of infectious diseases can be achieved when interventions are guided by the local epidemiology and accurate diagnostic tools. For melioidosis, data on serological responses to *B. pseudomallei* and their correlation with host determinants in humans is under-investigated. Understanding antibody responses to *B. pseudomallei* during infections is critical for the development of accurate serological diagnostics and effective vaccines. To date, most publications on epidemiological studies and diagnostics have reported antibody responses to crude antigens of *B. pseudomallei*. An indirect hemagglutination assay (IHA) is the most widely used serological test to detect antibodies against *B. pseudomallei*^7^. Despite its low diagnostic values, several studies have used IHA for epidemiology studies of melioidosis in Thailand and other areas due to the lack of a more accurate and simple test^8,9^. Other studies in Malaysia used culture filtrate antigen (CFA) for serology studies of septicemic melioidosis patients and suggest a role for different classes and subclasses of antibody responses in septicemic and localized melioidosis^10,11^. However, interpretation of results of these assays based on crude antigens can be complicated since such results represent the reactions of polyclonal antibodies against multiple antigens of *B. pseudomallei*.

Several assays based on purified antigens and recombinant proteins have been developed and evaluation has shown much improved performance in the diagnosis of melioidosis^12–16^. Recently, we demonstrated that rapid ELISAs based on detection of IgG to recombinant hemolysin co-regulated protein 1 (Hcp1) and O-polysaccharide (OPS) antigens of *B. pseudomallei* have diagnostic potential in melioidosis^13,14^. Hcp1 and OPS are two potential vaccine candidates^17,18^ and are known virulence factors of *B. pseudomallei*^19^. Hcp1 is located within the cluster 1 (also called cluster 5) type VI secretion system (T6SS) and plays a role in the bacterium’s intracellular lifestyle^19–22^. OPS is a component of lipopolysaccharide that is located on the bacterial outer membrane. These two antigens are highly recognized by melioidosis patient sera or plasma. The Hcp1-ELISA and OPS-ELISA have been successfully used for serological surveys of *B. pseudomallei* exposure in febrile patients in Myanmar^23^ and Cambodia^24^. Further, a rapid immunochromatography test based on Hcp1 (Hcp1-ICT) has been developed as a point-of-care (POC) test and evaluated for IgG detection in patients at risk for melioidosis in four hospitals in Thailand with significant improvement over IHA in identifying patients with melioidosis. The rapid ICT provides 88% sensitivity and 86–92% specificity compared with the culture method^16^. Thus, Hcp1 and OPS are potential targets for melioidosis diagnostics.

During infection, *B. pseudomallei* may induce different classes and subclasses of antibody responses to Hcp1 and OPS antigens. In theory, IgM is thought to be more representative of antibody for acute infection while IgG may represent a marker for convalescent and past infection. Different classes and subclasses of antibody facilitate diverse biological functions^25^. The prominent classes and subclasses of antibodies may influence the performance of the serological assays and confer some protective function in melioidosis. We hypothesized that IgA, IgM and IgG responses and IgG subclasses are induced differentially by Hcp1 and OPS antigens and may be correlated with characteristics of melioidosis patients.

In this study, we aimed to 1) determine the classes and subclasses of antibody responses to Hcp1 and OPS antigens in plasma samples from melioidosis patients and healthy donors, 2) assess the correlation between duration of clinical symptoms and classes and IgG subclasses of antibody responses in melioidosis patients, and 3) compare classes and subclasses of antibody responses between different groups of melioidosis patients (male versus female, DM versus non-DM, bacteremia versus non-bacteremia, survivors versus non-survivors, and renal disease versus non-renal disease). A collection of 102 plasma samples from melioidosis patients were used to characterize classes and IgG subclasses of antibody responses to OPS and Hcp1 antigens. This information may be useful for further development of serological assays for melioidosis. If specific antibody responses are associated with better outcomes, this may also provide useful information for vaccine development.

## Results

### Classes of antibody responses to Hcp1 and OPS in melioidosis patients and healthy donors

**Figure 1:**
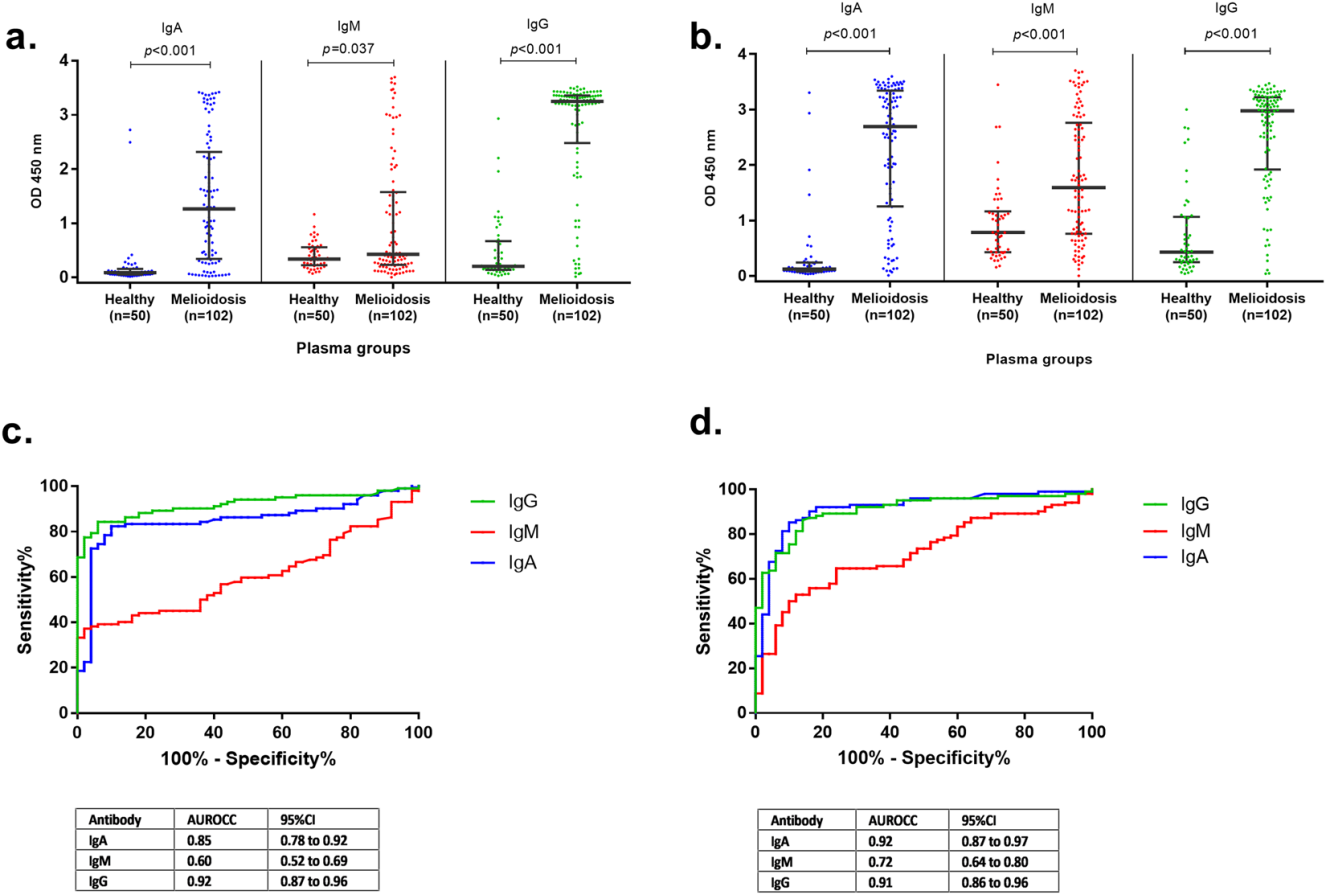
IgA, IgM and IgG antibodies to Hcp1 (**a**) and OPS (**b**) in plasma samples of healthy donors and melioidosis patients. ROC plots of IgA, IgM and IgG antibodies against Hcp1 (**c**) and OPS (**d**) in plasma from melioidosis patients versus healthy donors. The plasma samples were diluted at 1:250 for ELISAs.

We determined classes and subclasses of antibody responses against Hcp1 and OPS in a total of 152 plasma samples. These included culture-confirmed melioidosis patients (N = 102) and Thai healthy donors (N = 50). Quantitative results of OD values of antibodies against Hcp1-ELISA and OPS-ELISA were compared at plasma dilution of 1:250 and the results are shown in Fig. 1. We observed that the median optical density (OD) values of IgA, IgM and IgG responses to Hcp1 and OPS for melioidosis patients were significantly higher than for Thai healthy donors (P < 0.05 for all comparisons between melioidosis group versus Thai healthy donors group).

In the melioidosis group, anti-Hcp1 antibodies were characterized by high IgG but low IgA and IgM levels (Fig. 1a) in contrast to anti-OPS antibodies which were characterized by high levels of both IgA and IgG but low IgM levels (Fig. 1b). The median OD values of IgG, IgA and IgM against Hcp1 were 3.25 (IQR 2.48–3.36), 1.27 (IQR 0.34–2.32) and 0.43 (IQR 0.23–1.58), respectively. The median OD of IgG, IgA and IgM against OPS were 2.98 (IQR 1.92–3.22), 2.69 (IQR 1.25–3.34) and 1.59 (IQR 0.76–2.76), respectively. The median OD value of IgG against OPS was comparable to IgA [median OD 2.98 (IQR 1.92–3.22) versus 2.69 (IQR 1.25–3.34) (P = 0.613)].

In healthy donors, the median OD values of IgG, IgA and IgM against Hcp1 were low at 0.20 (IQR 0.14–0.67), 0.09 (IQR 0.05–0.16) and 0.34 (IQR 0.23–0.56), respectively. The median OD values of IgG, IgA and IgM against OPS were detected at 0.43 (IQR 0.25–1.06), 0.12 (IQR 0.08–0.24) and 0.78 (IQR 0.43–1.16), respectively.

We next compared the diagnostic potential of each antibody class in response to Hcp1 and OPS. Receiver operating characteristics (ROC) analyses of antibody responses were plotted by calculating the sensitivity and specificity of increasing number of true positive (% sensitivity) and false-positive rate (1 - % specificity). The results of ROC comparisons between classes of antibody responses to distinguish between melioidosis patients and Thai healthy donors are shown in Fig. 1c,d. Areas under receiver operating characteristic curve (AUROCC) of antibody responses to Hcp1 were highest for IgG (0.92) when compared to IgA (0.85) and IgM (0.60) (IgG versus IgA, P = 0.089; IgG versus IgM, P < 0.001; IgA versus IgM, P < 0.001) (Fig. 1c). When anti-OPS antibody classes were analyzed, AUROCCs of IgA were highest and comparable to IgG (0.92 versus 0.91), and the AUROCC of IgM was lowest (0.72) (IgG versus IgA, P = 0.465; IgG versus IgM, P < 0.001; IgA versus IgM, P < 0.001) (Fig. 1d).

### Subclasses of IgG responses to Hcp1 and OPS in melioidosis patients and healthy donors

**Figure 2:**
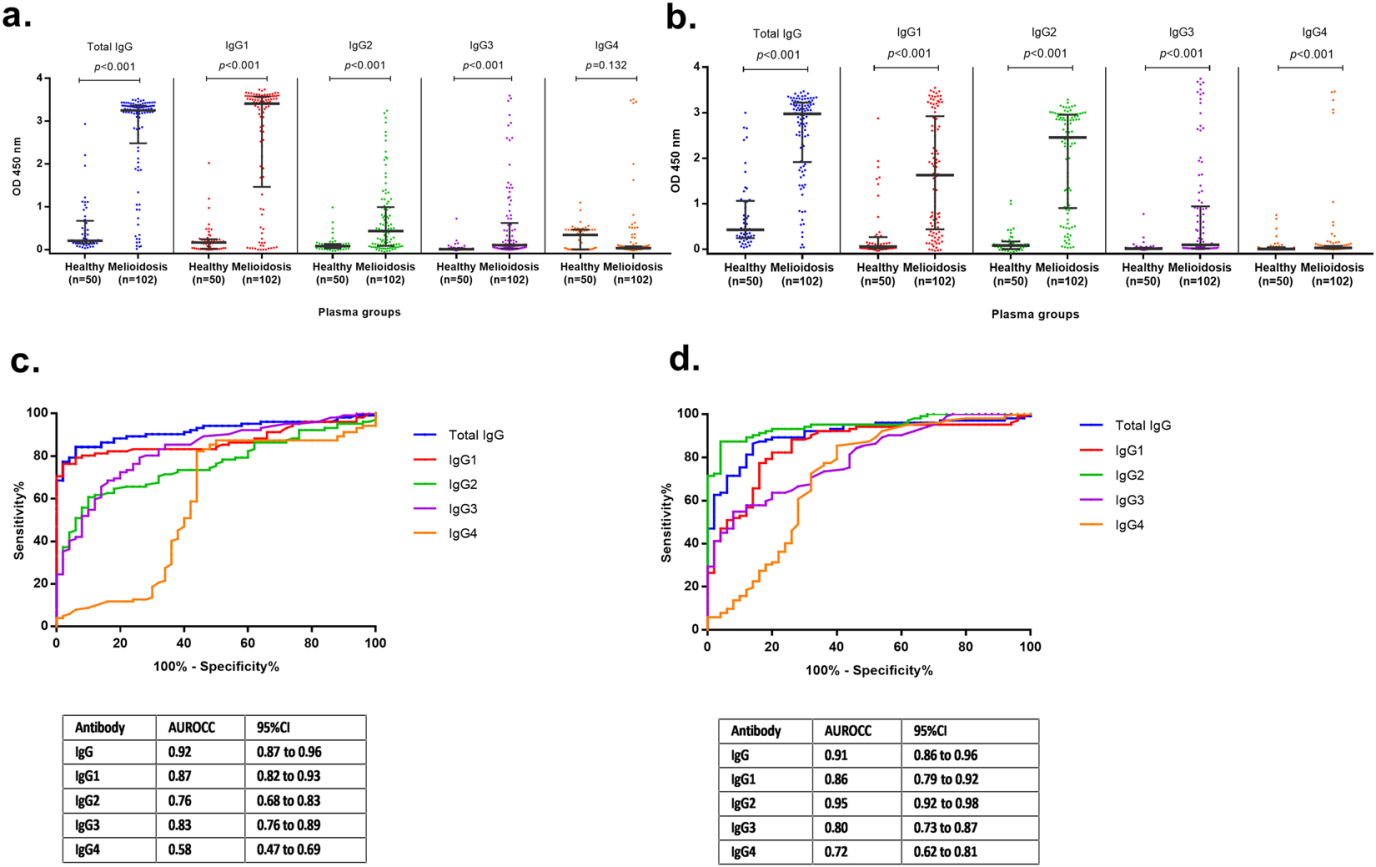
Total IgG and IgG subclasses of antibodies to Hcp1 (**a**) and OPS (**b**) in plasma samples of healthy donors and melioidosis patients. ROC plots of total IgG and IgG subclasses of antibodies against Hcp1 (**c**) and OPS (**d**) from melioidosis patients and healthy donors. The plasma samples were diluted at serum dilution 1:250 for ELISAs.

Quantitative results of OD values of total IgG and IgG subclasses of antibodies to Hcp1 and OPS are shown in Fig. 2. Different IgG subclasses against Hcp1 and OPS were detected in melioidosis patients. The results showed that high levels of IgG1 were induced by Hcp1 (Fig. 2a). In contrast, high levels of IgG2 and, to a lesser degree, IgG1 were induced by OPS (Fig. 2b). In melioidosis patients, the median OD values of IgG1, IgG2, IgG3 and IgG4 responses to Hcp1 were 3.41 (IQR 1.46–3.57), 0.43 (IQR 0.08–0.99), 0.10 (IQR 0.03–0.62), and 0.03 (IQR 0.01–0.08), respectively (Fig. 2a). The median OD values of IgG1, IgG2, IgG3 and IgG4 against OPS were 1.63 (IQR 0.44–2.92), 2.46 (IQR 0.91–2.96), 0.10 (IQR 0.02–0.94), and 0.03 (IQR 0.01–0.07), respectively (Fig. 2b). In healthy donors, the median OD values of IgG1, IgG2, IgG3, and IgG4 against Hcp1 were low at 0.16 (IQR 0.02–0.24), 0.08 (IQR 0.03–0.13), 0.01 (IQR 0.00–0.03), and 0.34 (0.00–0.47), respectively. The median OD values of IgG1, IgG2, IgG3, and IgG4 against OPS were 0.06 (IQR 0.02–0.27), 0.08 (IQR 0.00–0.17), 0.02 (IQR 0.01–0.03), and 0.01 (IQR 0.00–0.04), respectively. The median OD values of IgG1, IgG2 and IgG3 subclasses against both antigens for melioidosis patients were higher compared to Thai healthy donors (P < 0.05 for antibodies against Hcp1 and OPS for all comparisons between melioidosis patients and healthy donors).

The ROC analyses for distinguishing between melioidosis cases and healthy controls demonstrated AUROCCs of 0.87, 0.76, 0.83 and 0.58 for IgG1, IgG2, IgG3, IgG4 antibodies against Hcp1, respectively (Fig. 2c) and of 0.86, 0.95, 0.80 and 0.72 for IgG1, IgG2, IgG3, IgG4 antibodies against OPS, respectively (Fig. 2d). IgG2 subclass response to OPS showed the highest AUROCC (0.95) in comparison to other IgG subclasses (IgG2 versus IgG1, P = 0.012; IgG2 versus IgG3, P < 0.001; IgG2 versus IgG4, P < 0.001) (Fig. 2d) and the AUROCC of IgG2 was comparable to that of total IgG (IgG2 versus IgG, P = 0.177).

### Correlation of different classes and IgG subclasses of antibody response to Hcp1 and OPS in melioidosis patients

We determined the correlation of classes and subclasses of antibody responses to Hcp1 and OPS using serum samples from all melioidosis patients at day 0. The pairwise correlation coefficient (rho) of all antibodies against Hcp1 and OPS in plasma samples were analyzed and interpreted as previously described^26^. The results of antibodies against Hcp1 indicated low correlations in levels of IgM and IgA (rho = 0.45) and between IgM and total IgG (rho = 0.33). We found a moderate correlation between IgA and IgG (rho = 0.67, P < 0.001) and between IgG and IgG2 (rho = 0.65, P < 0.001) and a high correlation between total IgG and IgG1 (rho = 0.86, P < 0.001) (Supplementary Fig. S1a).

For antibody responses to OPS, the data showed high correlations between total IgG and IgG1 (rho = 0.73). Moderate correlations were observed between total IgG and IgG2 (rho = 0.67), between IgA and IgG (rho = 0.55, P < 0.001) and between IgA and IgM (rho = 0.53, P < 0.001) (Supplementary Fig. S1b).

We observed a low to moderate positive correlation of antibody responses between same classes or subclasses of antibody responses against Hcp1 versus OPS (IgA, rho = 0.49, P < 0.001; IgM, rho = 0.37, P < 0.001; IgG, rho = 0.34, P < 0.001; IgG1, rho = 0.41, *p* < 0.001; IgG2, rho = 0.48, *p* < 0.001; IgG3, rho = 0.24, P = 0.014; IgG4, rho = 0.69, P < 0.001) (Supplementary Fig. S1c).

### Effect of duration of clinical symptoms on antibody responses to Hcp1 and OPS

**Figure 3:**
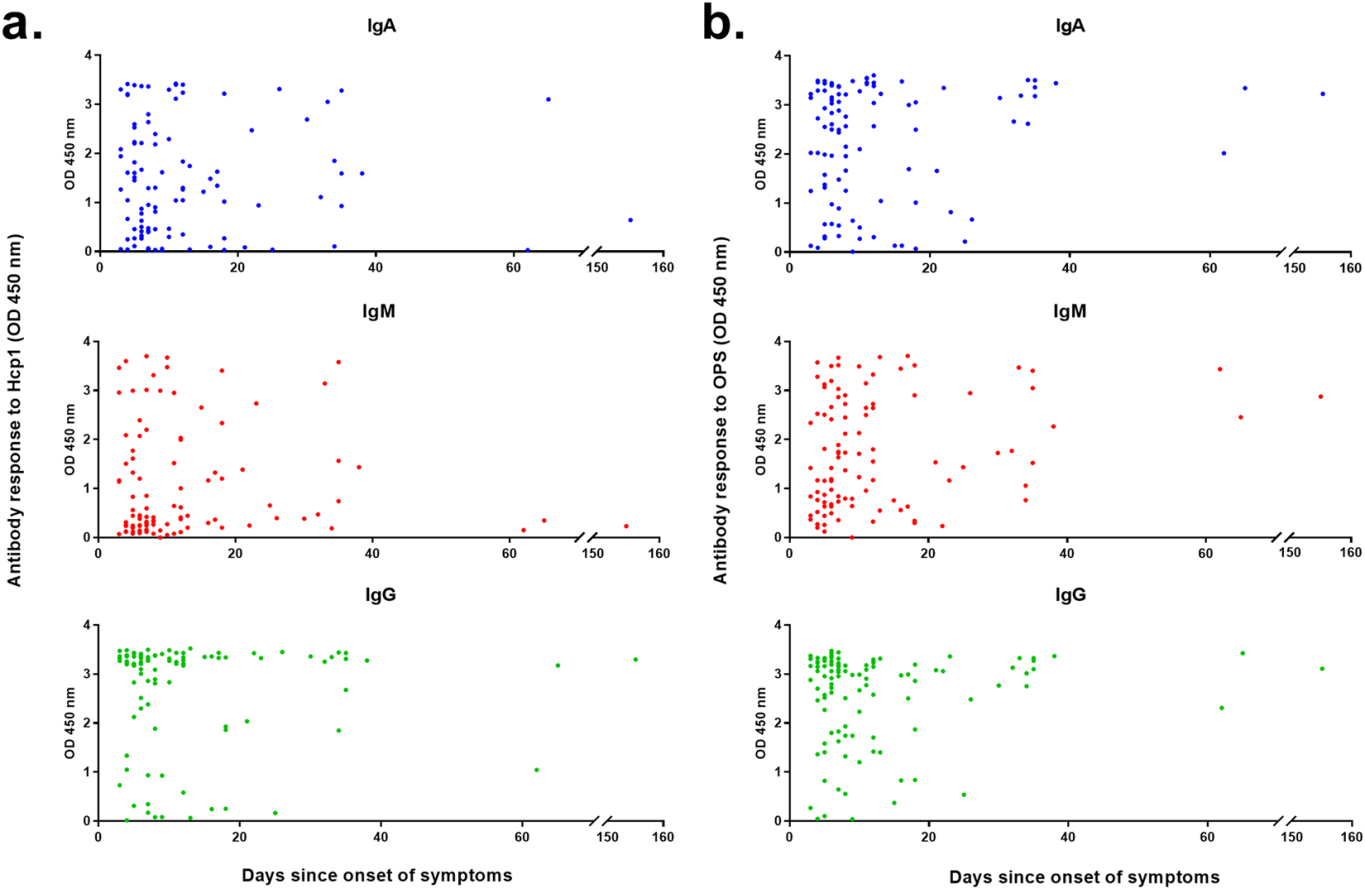
Effect of duration before admission on IgA, IgM, and IgG responses to Hcp1 (**a**) and OPS (**b**) in melioidosis patients. Aligned dot plots represent the OD.

We next analyzed the effect of duration of clinical symptoms on day 0 antibody responses to Hcp1 and OPS. The duration of clinical symptoms of melioidosis patients in our cohort ranged between 3–155 days, median 9 (IQR 6–16) days (Fig. 3). We detected significant IgA, IgM and IgG levels against both Hcp1 and OPS early in most of acute melioidosis patients although we observed some variations in antibody levels between individuals. For example, in 49 patients with symptom durations of 1–7 days, the median OD values of IgG and IgA against Hcp1 were higher than IgM [IgG 3.23 (IQR 2.67–3.36), IgA 1.05 (IQR 0.32–2.22) versus IgM 0.42 (IQR 0.21–1.56)] (Fig. 3a). The median OD values of IgG, IgA and IgM against OPS were all high 3.06 (IQR 2.37–3.26), 2.61 (IQR 1.28–3.26) and 1.19 (IQR 0.66–2.51), respectively (Fig. 3b).

### Effect of age on classes and subclasses of antibody responses to Hcp1 and OPS

The effect of age on day 0 classes and subclasses of antibody responses in melioidosis patients were analyzed using linear regression analysis. Scatter plots of the OD values of IgA, IgM and IgG responses to Hcp1 and OPS are shown in Supplementary Fig. S2 and P-values are shown in Table 1. The age range of our melioidosis cohort was 19–91 years [median 56 (IQR 46–64) years]. We observed no relationship between age and median OD values of IgA or IgM antibodies against Hcp1 nor between age and median OD values of IgA or IgG or IgM antibodies against OPS (Table 1 and Supplementary Fig. S2).

**Table 1:** Classes and subclasses of antibody responses to Hcp1 and OPS in different groups of melioidosis patients.

Variable		Median (IQR) of OD450 of antibody responses to Hcp1														
		Number (%)	IgA	P-value	IgM	P-value	IgG	P-value	IgG1	P-value	IgG2	P-value	IgG3	P-value	IgG4	P-value
Age		102 (100%)		0.798^a^		0.703^a^		**0.009** ^a^		**0.045** ^a^		0.191^a^		0.682^a^		0.072^a^
Gender	Male	76 (77.5%)	1.13 (0.33–2.23)	0.415^b^	0.42 (0.24–1.37)	0.592^b^	3.22 (2.55–3.37)	0.595^b^	3.35 (1.56–3.56)	0.721^b^	0.40 (0.08–0.90)	0.676^b^	0.09 (0.03–0.69)	0.986^b^	0.03 (0.01–0.08)	0.566^b^
	Female	26 (26.5%)	1.40 (0.38–3.20)	0.321^c^	0.45 (0.21–2.00)	0.722^c^	3.27 (1.66–3.33)	0.678^c^	3.51 (0.81–3.58)	0.869^c^	0.49 (0.08–1.27)	0.398^c^	0.12 (0.04–0.53)	0.768^c^	0.04 (0.01–0.15)	0.945^c^
Diabetes mellitus	Diabetic	66 (67.3%)	1.32 (0.45–2.42)	0.352^b^	0.46 (0.25–2.00)	0.127^b^	3.26 (2.78–3.36)	0.541^b^	3.43 (1.64–3.57)	0.448^b^	0.49 (0.12–1.13)	0.200^b^	0.12 (0.03–1.16)	0.114^b^	0.03 (0.01–0.07)	0.949^b^
	Non-Diabetic	36 (36.7%)	0.91 (0.27–2.15)	0.452^c^	0.37 (0.16–1.16)	0.195^c^	3.23 (2.06–3.36)	0.385^c^	3.22 (0.51–3.58)	0.505^c^	0.17 (0.06–0.90)	0.821^c^	0.06 (0.02–0.39)	0.108^c^	0.03 (0.01–0.10)	0.235^c^
Bacteremia	Bacteremia	91 (92.8%)	1.27 (0.40–2.21)	0.875^b^	0.44 (0.24–1.77)	0.207^b^	3.25 (2.68–3.36)	0.842^b^	3.42 (1.68–3.56)	0.934^b^	0.39 (0.08–0.98)	0.463^b^	1.10 (0.03–0.58)	0.615^b^	0.03 (0.01–0.08)	0.951^b^
	Non-bacteremia	11 (11.2%)	1.85 (0.10–2.64)	0.807^c^	0.26 (0.19–0.60)	0.149^c^	3.24 (1.34–3.36)	0.486^c^	3.27 (0.16–3.63)	0.526^c^	0.63 (0.19–1.33)	0.853^c^	0.09 (0.01–0.90)	0.924^c^	0.03 (0.02–0.07)	0.410^c^
Renal disease	Renal disease	14 (13.7%)	0.79 (0.06–1.93)	0.215^b^	0.40 (0.18–0.74)	0.251^b^	3.16 (0.97–3.36)	0.565^b^	2.94 (0.16–3.51)	0.255^b^	0.10 (0.02–0.45)	**0.030** ^b^	0.02 (0.00–0.08)	**0.001** ^b^	0.02 (0.01–0.17)	0.555^b^
	Non-renal disease	88 (86.3%)	1.30 (0.41–2.45)	0.264^c^	0.45 (0.24–1.94)	0.125^c^	3.25 (2.71–3.36)	0.280^c^	3.43 (1.75–3.58)	0.199^c^	0.49 (0.12–1.06)	0.226^c^	0.12 (0.04–0.80)	**0.047** ^c^	0.04 (0.02–0.08)	0.100^c^
28-day mortality	Survived	54 (55.1%)	1.47 (0.26–2.23)	0.975^b^	0.46 (0.23–1.48)	0.801^b^	3.25 (1.99–3.34)	0.403^b^	3.45 (0.33–3.55)	0.835^b^	0.47 (0.06–1.10)	0.985^b^	0.21 (0.04–0.94)	**0.015** ^b^	0.04 (0.01–0.08)	0.850^b^
	Died	48 (49.0%)	1.04 (0.36–2.54)	0.975^c^	0.39 (0.24–2.04)	0.891^c^	3.25 (2.71–3.38)	0.206^c^	3.30 (1.75–3.58)	0.583^c^	0.40 (0.11–0.92)	0.592^c^	0.07 (0.02–0.13)	0.196^c^	0.03 (0.01–0.08)	0.451^c^
**Variable**		**Median (IQR) of OD450of antibody responses to OPS**														
		**Number (%)**	**IgA**	**P-value**	**IgM**	**P-value**	**IgG**	**P-value**	**IgG1**	**P-value**	**IgG2**	**P-value**	**IgG3**	**P-value**	**IgG4**	**P-value**
Age		102 (100%)		0.734^a^		0.543^a^		0.074^a^		0.250^a^		0.079^a^		0.339^a^		**0.036** ^a^
Gender	Male	76 (77.5%)	2.59 (0.91–3.29)	**0.047** ^b^	1.28 (0.63–2.49)	**0.005** ^b^	2.91 (1.76–3.18)	0.062^b^	1.71 (0.41–2.88)	0.757^b^	2.53 (0.86–2.97)	0.935^b^	0.10 (0.02–0.91)	0.716^b^	0.03 (0.02–0.08)	0.226^b^
	Female	26 (26.5%)	3.14 (2.08–3.39)	**0.024** ^c^	2.58 (1.37–3.06)	**0.008** ^c^	3.12 (2.65–3.31)	0.099^c^	1.03 (0.51–3.12)	0.666^c^	2.33 (1.20–2.92)	0.782^c^	0.15 (0.03–1.26)	0.619^c^	0.02 (0.01–0.05)	0.615^c^
Diabetes mellitus	Diabetic	66 (67.3%)	2.97 (1.98–3.39)	**0.004** ^b^	1.72 (0.87–2.88)	0.118^b^	3.04 (2.30–3.22)	0.202^b^	1.82 (0.59–2.92)	0.307^b^	2.61 (1.28–2.97)	0.106^b^	0.11 (0.02–1.08)	0.340^b^	0.04 (0.02–0.07)	0.329^b^
	Non-Diabetic	36 (36.7%)	1.66 (0.55–3.13)	**0.002** ^c^	1.33 (0.63–2.44)	0.142^c^	2.78 (1.33–3.22)	0.077^c^	1.16 (0.26–2.98)	0.322^c^	2.33 (0.49–2.85)	0.112^c^	0.08 (0.02–0.59)	0.444^c^	0.02 (0.01–0.09)	0.276^c^
Bacteremia	Bacteremia	91 (92.8%)	2.61 (1.25–3.29)	0.331^b^	1.64 (0.76–2.87)	0.353^b^	2.98 (1.83–3.23)	0.956^b^	1.56 (0.39–2.87)	0.226^b^	2.37 (0.87–2.92)	0.172^b^	0.09 (0.02–1.03)	0.688^b^	0.03 (0.01–0.07)	0.296^b^
	Non-bacteremia	11 (11.2%)	3.14 (0.89–3.46)	0.551^c^	1.17 (0.76–1.89)	0.276^c^	2.95 (2.75–3.15)	0.540^c^	2.01 (0.48–3.29)	0.280^c^	2.89 (2.44–2.97)	0.132^c^	0.10 (0.02–0.41)	0.241^c^	0.04 (0.02–0.10)	0.554^c^
Renal disease	Renal disease	14 (13.7%)	1.60 (0.24–3.31)	0.057^b^	0.78 (0.42–1.82)	**0.043** ^b^	2.73 (1.18–3.09)	0.144^b^	1.52 (0.44–2.83)	0.965^b^	1.81 (0.32–2.77)	0.114^b^	0.02 (0.01–0.30)	0.075^b^	0.02 (0.01–0.05)	0.310^b^
	Non-renal disease	88 (86.3%)	2.80 (1.50–3.34)	0.021^c^	1.72 (0.84–2.87)	**0.042** ^c^	2.99 (2.24–3.24)	0.176^c^	1.63 (0.41–2.92)	0.904^c^	2.58 (0.98–2.97)	0.168^c^	0.10 (0.02–1.06)	0.238^c^	0.03 (0.02–0.08)	0.097^c^
28-day mortality	Survived	54 (55.1%)	2.78 (1.34–3.37)	0.501^b^	1.43 (0.67–2.65)	0.271^b^	2.98 (2.30–3.28)	0.091^b^	1.73 (0.35–3.11)	0.818^b^	2.60 (1.01–2.97)	0.208^b^	0.08 (0.02–0.73)	0.632^b^	0.04 (0.02–0.08)	0.730^b^
	Died	48 (49.0%)	2.59 (0.92–3.22)	0.555^c^	1.96 (0.81–2.90)	0.226^c^	2.95 (1.76–3.19)	0.479^c^	1.58 (0.49–2.84)	0.746^c^	2.39 (0.59–2.86)	0.408^c^	0.19 (0.02–1.54)	0.363^c^	0.03 (0.01–0.06)	0.107^c^

Abbreviations: a: P-value (linear regression); b: P-value (Mann-Whitney test); c: P-value (logistic regression); d: P-value (ANOVA).

### Longitudinal analysis of classes and subclasses of antibody responses to Hcp1 and OPS

**Figure 4:**
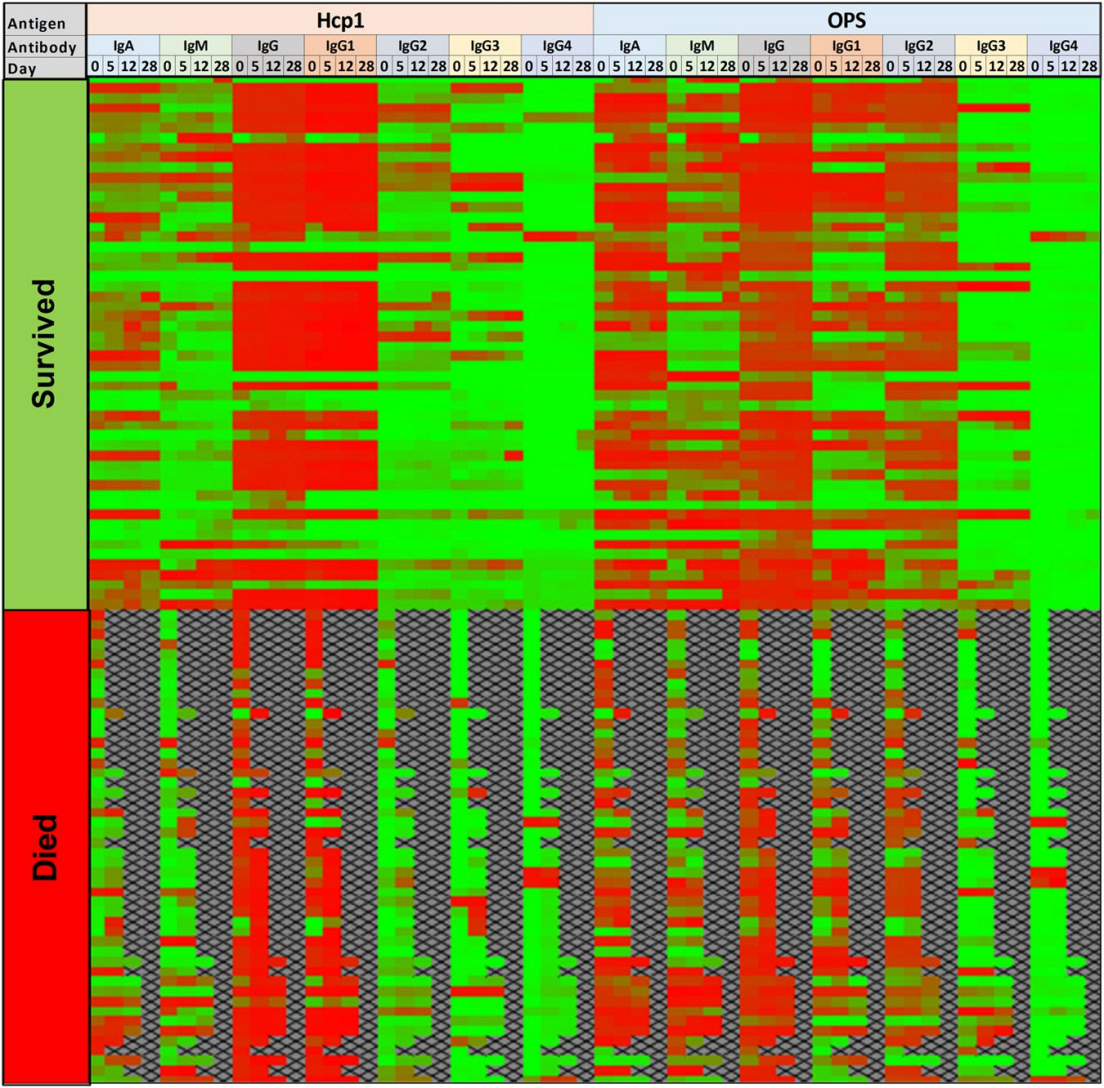
Heat map of longitudinal analysis of classes and subclasses of antibody responses to Hcp1 and OPS in plasma samples of survivors and non-survivors melioidosis. The antibody levels were determined at day 0, day 5, day 12 and day 28 by ELISAs using plasma dilution 1:250. Red represents high OD and green represnts low OD.

Antibody responses were analyzed in plasma samples collected from melioidosis patients at day 0, day 5, day 12 and day 28 of enrolment, stratified by survival to 28 days. The heat map results of longitudinal analysis of classes and subclasses of antibody against Hcp1 and OPS in survivors and non-survivors at 28-day mortality are shown in Fig. 4. These data confirmed that *B. pseudomallei* Hcp1 strongly elicited IgG, predominantly IgG1 responses at day 0, day 5, day 12 and day 28, whilst *B. pseudomallei* OPS induced more various classes of antibody response, which included high levels of IgA, IgG but a lower level of IgM. IgG2 was the predominant IgG subclass, which responded to OPS (Fig. 4). The OD values of antibodies against Hcp1 in individuals showed high and persistent levels of these antibodies at all days. However, we observed that the OD values of antibodies against OPS in some individuals gradually increased at day 5, day 12 and day 28.

Scatter plots of OD values for longitudinal analysis of classes and subclasses of antibody responses to Hcp1 and OPS in plasma samples of melioidosis patients are demonstrated in Supplementary Figs S3 and S4, respectively. At day 0, the median OD value of IgG3 response to Hcp1 in survivors (N = 54) was significantly higher than in non-survivors to 28 days (N = 48) (0.21 (IQR 0.04–0.94) versus 0.07 (IQR 0.02–0.13), P = 0.015) (Table 1 and Supplementary Fig. S3). The median OD values of all classes and IgG subclasses antibodies against Hcp1 other than IgG3 in survivors were not different compared to non-survivors.

In survivors, the median OD value of IgA antibody against Hcp1 was increased from 1.47 (IQR 0.26–2.23) at day 0 to 1.69 (IQR 0.67–2.53) at day 5 and to 1.72 (IQR 0.73–2.67) at day 12; IgM antibody was slightly increased from 0.46 (IQR 0.23–1.48) at day 0 to 0.64 (IQR 0.31–1.71) at day 5 and to 0.96 (IQR 0.35–2.19) at day 12 while total IgG and IgG1 antibodies were very high at all time points with median OD values at day 0, day 5, day 12, day 28 = 3.45 (IQR 0.33–3.55), 3.51 (IQR 0.59–3.57), 3.54 (IQR 0.89–3.60) and 3.54 (IQR 0.66–3.66), respectively. IgG2 against Hcp1 were lower than IgG1 and did not demonstrate significant changes at day 12 and day 28.

The median OD values of IgA, IgM and IgG antibodies to OPS at day 0 were not significantly different between survivors and non-survivors to 28 days (Table 1 and Supplementary Fig. S4). We observed that the median OD values of IgA, IgM, IgG1 and IgG2 against OPS of survivors group were increased at day 5 and day 12. The median OD value of IgA antibody against OPS was increased from 2.78 (IQR 1.34–3.37) at day 0 to 3.09 (IQR 1.95–3.39) at day 5 and to 3.13 (IQR 2.12–3.41) at day 12; IgM was increased from 1.43 (IQR 0.67–2.65) at day 0 to 1.84 (IQR 0.87–3.15) at day 5 and to 2.33 (IQR 0.99–3.31) at day 12; IgG1 was increased from 1.73 (IQR 0.35–3.11) at day 0 to 2.13 (IQR 0.37–3.22) at day 5 and to 2.28 (IQR 0.60–3.26) at day 12 and IgG2 was increased from 2.60 (IQR 1.01–2.97) at day 0 to 2.86 (IQR 1.68–2.94) at day 5 and to 2.86 (IQR 0.08–3.01) at day 12. The median OD values of IgG3 and IgG4 were low and did not show increased levels at day 5 and day 12.

Interestingly, the median OD of IgG2 responses to OPS were significantly lower in non-survivors with early death (died within 3 days) compared with survivors [1.66 (IQR 0.41–2.46) versus 2.60 (IQR 1.01–2.97), P = 0.037] (Supplementary Fig. S4). In contrast, the median OD values of IgA, IgM, IgG, IgG1, IgG3, and IgG4 responses to OPS in the survivors group were not statistically different from non-survivors group (died within 3 days) (Supplementary Fig. S4).

### Antibody responses to Hcp1 and OPS in melioidosis patients with bacteremia

We next compared the antibody responses between the bacteremia group (N = 91) and non-bacteremia group (N = 11) of melioidosis patients. The median OD values of antibodies to Hcp1 and OPS were analyzed using plasma samples collected at day 0. The median OD data in Table 1 and Supplementary Fig. S5 demonstrate that there were no significant differences in levels of all classes and IgG subclasses of antibodies against Hcp1 and OPS between the bacteremia and non-bacteremia groups at day 0. A longitudinal heat map of classes and subclasses of antibodies against Hcp1 and OPS in bacteremic and non-bacteremic patients is shown in Supplementary Fig. S6.

### Effect of gender on classes and subclasses of antibody responses to Hcp1 and OPS

To investigate the effect of gender on antibody responses in melioidosis, we next determined IgA, IgM and IgG responses to Hcp1 and OPS antigens in male (N = 76) and female (N = 26) patients (Table 1 and Supplementary Fig. S7). We observed no difference in median OD values of all classes and subclasses of antibody responses to Hcp1 between the two genders (P > 0.05 for all antibodies). In contrast, the median OD values of IgA and IgM responses to OPS were significantly higher in females compared to males [IgA, 3.14 (IQR 2.08–3.39) versus 2.59 (IQR 0.91–3.29), P = 0.047; IgM, 2.58 (1.37–3.06) versus 1.28 (0.63–2.49), P = 0.005] (Table 1 and Supplementary Fig. S7). Logistic analyses confirmed significant differences in IgA and IgM responses to OPS between the two genders (P = 0.024 for IgA and P = 0.008 for IgM) (Table 1).

### Antibody responses to Hcp1 and OPS in diabetic and non-diabetic melioidosis patients

We compared the classes and subclasses of antibody responses between diabetic melioidosis patients (N = 66) and non-diabetic melioidosis patients (N = 36). The median OD values of IgA, IgM, IgG and all IgG subclasses responses to Hcp1 of diabetic patients were not statistically different from non-diabetic patients (Table 1 and Supplementary Fig. S8). Interestingly, the median OD value of IgA response to OPS for diabetic patients was significantly higher than for non-diabetic patients (2.97 (IQR 1.98–3.39) versus 1.66 (IQR 0.55–3.13), P = 0.004) but the median OD values of IgM and IgG and all IgG subclasses for the diabetic melioidosis group were not significantly different from non-diabetic melioidosis group (Table 1 and Supplementary Fig. S8).

**Figure 5:**
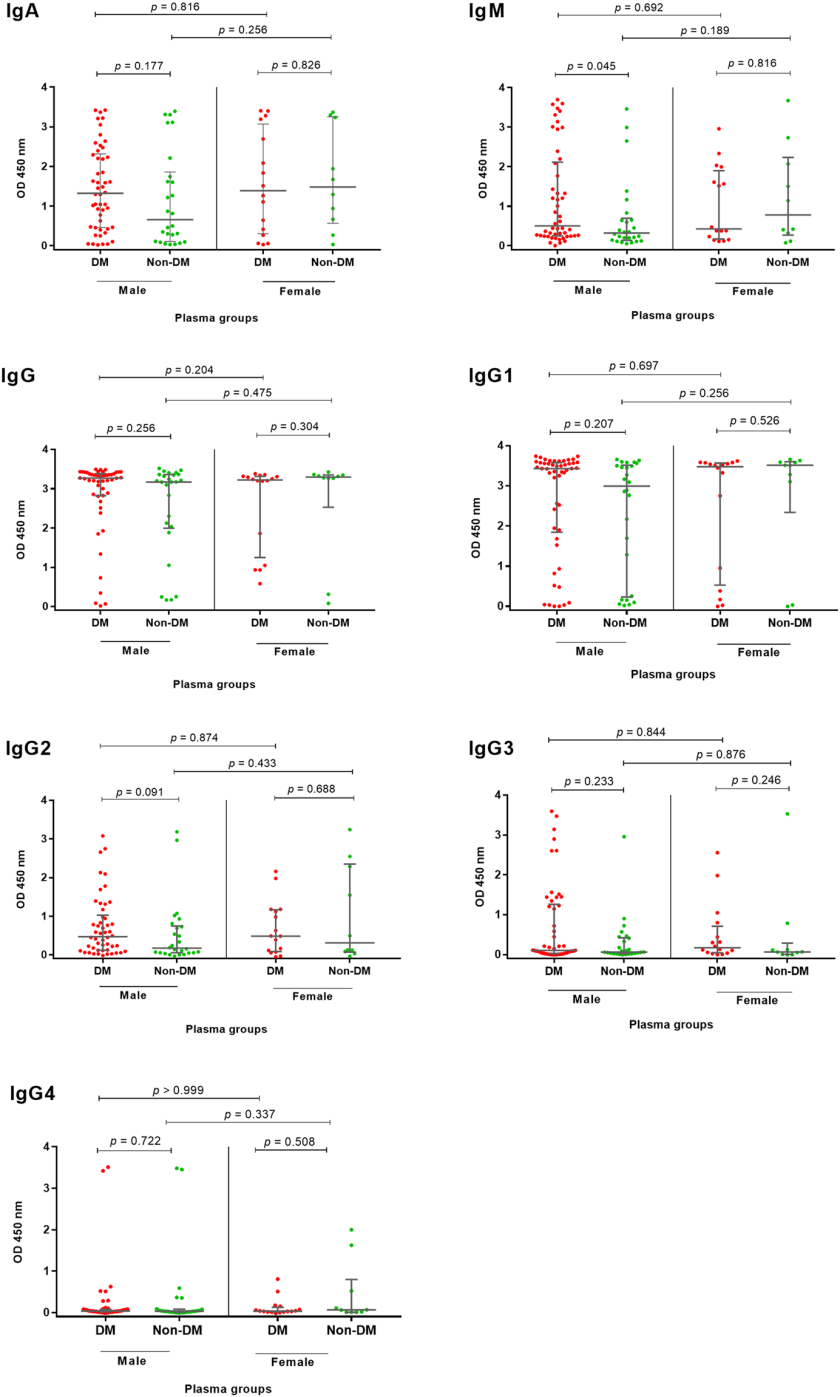
Antibody response to Hcp1 in diabetic and non-diabetic melioidosis patients and compare between male and female. Antibodies against Hcp1 were determined by ELISAs using plasma samples from melioidosis patients at dilution 1:250. Scatter plots represent antibody levels of individual patients. Median line and 25^th^ and 75^th^ percentile boundaries are shown.

**Figure 6:**
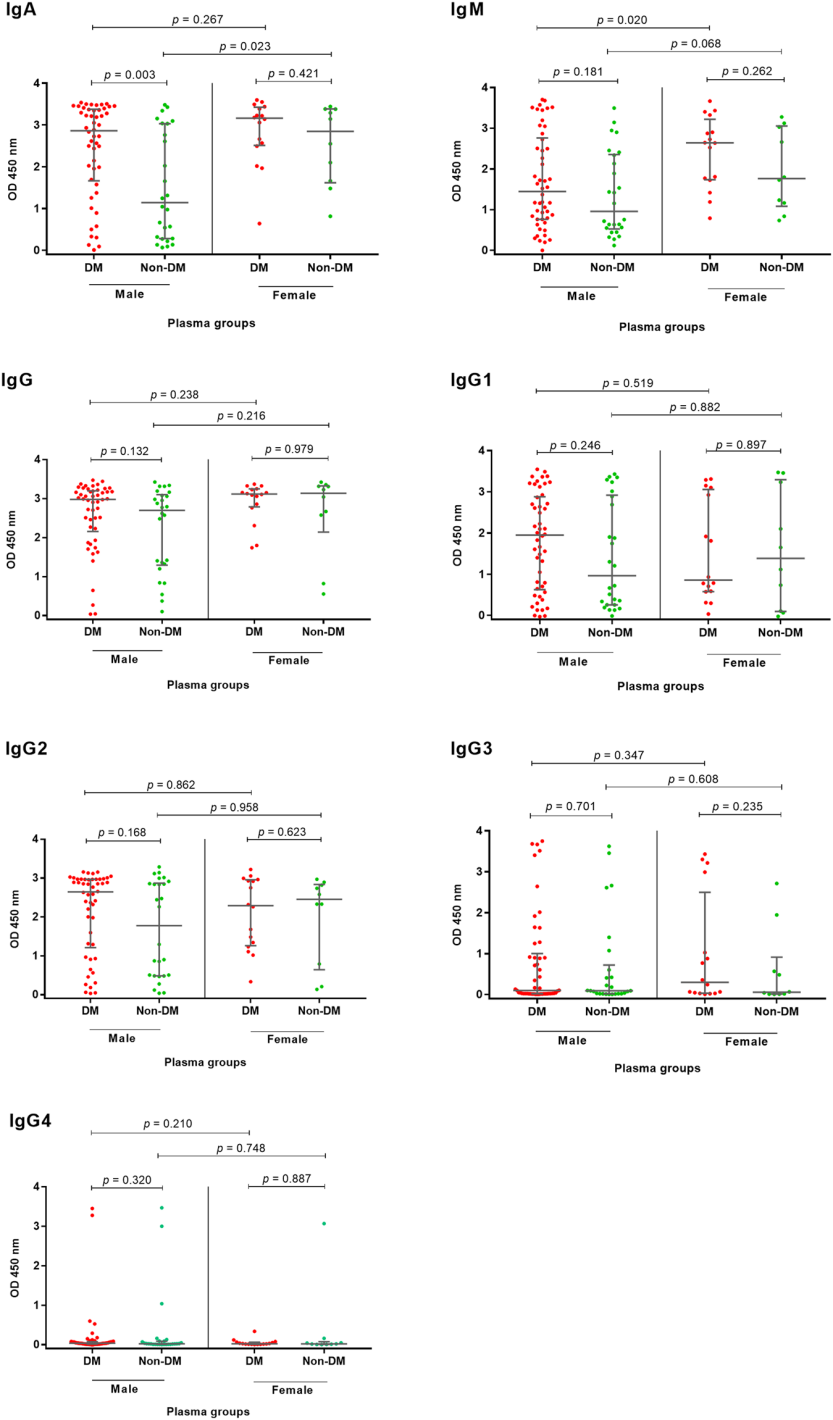
Antibody response to OPS in diabetic and non-diabetic melioidosis patients and compare between male and female. Antibodies against OPS were determined by ELISAs using plasma samples from melioidosis patients at dilution 1:250. Scatter plots represent antibody levels of individual patients. Median line and 25^th^ and 75^th^ percentile boundaries are shown.

We considered that the observed differences in antibody responses in diabetic melioidosis patients could be a confounding effect of gender. Therefore, we compared median OD values of antibody classes and subclasses between diabetes and non-diabetes within male or female groups. We observed a difference between the two groups for IgM against Hcp1 in the male group but not for other antibodies (Fig. 5). We also found a significant difference for IgA against OPS in the male group (N = 76), but not the female group (Fig. 6). Interestingly, the median OD value of IgA in diabetic melioidosis patients (N = 50) was significantly higher than non-diabetic melioidosis patients (N = 26) [2.86 (IQR 1.66–3.38) versus 1.14 (IQR 0.28–3.03), P = 0.003] (Fig. 6). In female groups (N = 26), the median OD value was not significantly different between diabetic (N = 16) and non-diabetic melioidosis patients (N = 10) [3.16 (IQR 2.51–3.43) versus 2.85 (IQR 1.62–3.38), P = 0.421).

The IgA levels against OPS were also compared between male and female melioidosis patients within diabetic or non-diabetic groups. In diabetes groups (N = 66), we found no difference in the median OD value of IgA between male patients (N = 50) and female patients (N = 16) [2.86 (IQR 1.66–3.38) versus 3.16 (IQR 2.51–3.43), P = 0.267] (Fig. 6). However, in non-diabetic group (N = 36), we observed that the median IgA (IQR) was significantly lower in male patients (N = 26) compared with female patients (N = 10) [1.14 (IQR 0.28–3.03) versus 2.85 (IQR 1.62–3.38), P = 0.023].

### Antibody responses to Hcp1 and OPS in melioidosis patients with renal disease and non-renal disease

Chronic renal disease is often reported in patients with melioidosis. We compared the classes and subclasses of antibody responses between melioidosis patients with pre-existing renal disease (N = 14) and non-renal disease (N = 88). The median OD values of IgA, IgM, IgG, IgG1 and IgG4 responses to Hcp1 of renal disease patients were not statistically different from non-renal disease patients (Table 1). However, we found the median OD values of IgG2 and IgG3 responses to Hcp1 for renal patients were significantly lower than for non-renal patients [IgG2, 0.10 (IQR 0.02–0.45) versus 0.49 (IQR 0.12–1.06), P = 0.030; IgG3, 0.02 (IQR 0.00–0.08) versus 0.12 (IQR 0.04–0.80), P = 0.001].

The median OD value of IgM responses to OPS of renal disease patients were statistically lower than non-renal disease patients [1.72 (IQR 0.84–2.87) versus 0.78 (IQR 0.42–1.82), P = 0.043]. The median OD values of IgA, IgG and all IgG subclasses responses to OPS for non-renal disease patients were not significantly different from renal disease patients (Table 1).

## Discussion

Hcp1 and OPS expressed by *B. pseudomallei* are potential diagnostic antigens and vaccine candidates for melioidosis^16–18^. In this study, we used established ELISAs to determine classes and IgG subclasses of specific antibody responses to Hcp1 and OPS in plasma samples from 102 melioidosis patients and 50 healthy donors. Our data demonstrate that these two antigens elicited distinct classes and subclasses of specific antibodies in melioidosis patients. The IgG antibody levels for both antigens were high in most patients at early time points. We observed different correlations in antibody levels between different classes and different IgG subclasses in recognizing the same antigen. We also noted different correlations in antibody levels between the same classes and IgG subclasses recognizing the different antigens. Our data suggest that gender, renal disease and diabetic status of infected patients can influence classes and subclasses profiles of antibody responses to Hcp1 and OPS antigens.

Our previous studies reported that Hcp1 and OPS are promising candidates for serodiagnosis of melioidosis in endemic areas^14,16,27^. The present study analyzing a different melioidosis cohort confirmed and extended the findings in the previous study. Analysis of antibody profiles of melioidosis patients at enrolment suggests that natural human antibody responses to Hcp1 are primarily restricted to IgG1 while antibodies specific to OPS of *B. pseudomallei* showed a broader range of responses with high levels of IgG2 followed by IgG1 and IgA. This is probably due to the fact that Hcp1 is a T-cell dependent protein antigen. This has been supported by a previous study showing that Hcp1 is associated with MHC class II on the surface of antigen presenting cells^21^. Hcp1 is a component of a virulence associated T6SS which plays a role in the intracellular lifestyle of *B. pseudomallei*. It is expressed inside of host cells and is involved in multinucleated giant cell (MNGC) formation^21,29,30^. Data obtained in the current study indicate that Hcp1 is highly immunogenic and stimulates antibody responses in humans. The isotype restriction results for Hcp1 suggest that serological tests based on total IgG predominantly measures IgG1 subclass.

Several studies have reported that human IgG responses to carbohydrate antigens of bacteria are primarily restricted to the IgG2 subclass^31^ which is also consistent with the results of the antibody responses to OPS in this study. Total plasma IgG levels may vary considerably between individual patients but the median ODs of IgG subclasses was highest for IgG2 followed by IgG1. It is likely that the OPS component of *B. pseudomallei* lipopolysaccharide activates humoral immune response in a T-cell independent manner^28^. Our results indicate more variation in classes and subclasses of OPS-specific antibodies compared to Hcp1-specific antibodies, which suggests that in a serological assay based on OPS multiple classes and IgG subclasses are detected. In addition, ROC analyses for distinguishing between melioidosis patients and healthy donors revealed the highest AUROCC for IgG2 against OPS (AUROCC = 0.95) and was comparable to total IgG against Hcp1 (AUROCC = 0.92). In comparison to other classes and subclasses of antibodies, the results in this study suggest that measurement of IgG2 antibodies against OPS rather than total IgG may not improve the diagnostic accuracy for the assay based on OPS.

Our comparison of AUROCCs showed that IgM antibodies to both Hcp1 and OPS has provided lowest diagnostic value when used in endemic areas for melioidosis. The data in this study indicated that IgM was the most cross-reactive class, which is in accordance with the literature from other bacterial infections^32^. IgM might be considered a part of innate immunity as well as natural antibodies^33^. In contrast, ROC analysis also demonstrated that detection of plasma IgA responses to Hcp1 and OPS were more specific to melioidosis than IgM for distinguishing between melioidosis and healthy donors in northeast Thailand. Interestingly, this study demonstrates significant levels of IgA recognizing both antigens although the median ODs of IgA were significantly lower than IgG. IgA can be present at the mucosal surfaces where interaction between pathogen and host occurs and may be considered as an innate immune effector molecule^33^. *B. pseudomallei* can infect humans and animals by inhalation, ingestion and inoculation^4,34^. The increased in levels of plasma IgA in melioidosis patients might be a result of interaction between *B. pseudomallei* and mucosal surfaces of the respiratory and gastrointestinal tracts^35,36^.

Correlation analysis consistently showed IgG responses specific to Hcp1 or OPS were more correlated with IgA compared to IgM, which would indicate a greater specificity of IgA than IgM. This specific IgA and IgG might represent the fraction of switched IgA and IgG respectively from IgM^37^. The increased avidity of antibodies could occur during the switching process^25^. Correlation of same classes and same IgG subclasses of the two different antigens were low to moderate and support the data shown earlier that different pathways of humoral immune responses may be used in recognizing Hcp1 and OPS antigens from the same organism.

The duration of symptoms in the melioidosis patients in this study ranged from 3–155 days indicating that the antibody levels detected in individual patients may represent variable durations of time after seroconversion. Melioidosis patients have varied clinical symptoms and *B. pseudomallei* can remain latent for a long period before melioidosis develops^4^. The high variation in IgA and IgM levels for both OPS and Hcp1 did not support the idea to use IgM as a diagnostic for acute infection. Instead, our results indicate that IgG responses were more significant in individual patients with both short and long duration of symptoms.

IgG antibody responses to LPS of *B. pseudomallei* has been previously shown to be associated with survival in melioidosis^38^. Our heat map analysis provides longitudinal data of individuals which demonstrate high variability in classes and subclasses of antibody responses to Hcp1 and OPS between individual patients, although the overall data indicated high and persistent levels of IgG and IgG1 antibodies against Hcp1 in majority of the patients, and showed that the IgA, IgM and IgG antibodies against OPS of some patients could increase at convalescent periods. However, some patients may have low or undetectable levels of these antibodies over time. Many host factors may affect the different antibody profiles including immunodeficiency^39^. When 3-day mortality was analyzed, the data suggested that IgG3 against Hcp1 and IgG2 against OPS may be involved in improving the outcome of the patients.

Females and males differ in the energy consumption and nutritional requirements which are based on the interactions between environmental factors and sex hormones^40^. Interestingly, our data suggest that female melioidosis patients produce significantly higher IgA and IgM antibodies against OPS than male patients. Previous studies suggest that females have enhanced capability of producing antibody to mount more effective resistance to several infections^40–42^. This may be due to female sex hormones which impact microbial composition and the resulting immune response via secondary metabolites binding with receptors like estrogen receptors and peroxisome proliferator-activated receptors^43^. Melioidosis more commonly affects males than females, with a male-to-female incidence ratio of 3:2 in Thailand^44^. It is possible that the differences in immune responses between genders and in environmental exposure may lead to variability in acquiring *B. pseudomallei* infection and severity of the disease.

Our previous study demonstrated higher seropositivity for total IgG against both Hcp1 and OPS in diabetic melioidosis patients compared to those without diabetes^14^. However, in this study there was no significant difference in the median OD of IgG levels for Hcp1 and OPS between the two groups. This may reflect other patient-level differences that we did not consider and further study of the diabetes-specific immune response to melioidosis is required. Higher median OD of IgA against OPS for the diabetic melioidosis group compared to non-diabetic melioidosis group was observed. One possible explanation for this finding is the enhancement of B-cell stimulation in type 2 diabetes secondary to chronic hyperactivation of mucosal immunity in response to *B. pseudomallei* infection^45^. When gender and diabetic status of melioidosis patients were considered separately, the data clearly demonstrate that both gender and diabetes were independently associated with IgA levels against OPS. In female melioidosis patients, the enhancement of IgA against OPS appeared in both diabetic and non-diabetic female groups. However, in the male group, only diabetic patients induced a more intense IgA response to the OPS of *B. pseudomallei*. Thus, our data suggest that synergistic action of being female and of diabetes can contribute to the profile of human antibody responses to *B. pseudomallei*. Future studies are required to explore the causality and exact mechanisms of the difference in antibody levels to *B. pseudomallei* in different genders and in diabetics versus non-diabetics.

The data in this study indicated that melioidosis patients with renal disease had lower median OD values for IgG2 and IgG3 against Hcp1 and IgM against OPS compared to patients without renal disease. Chronic kidney disease is associated with immune dysfunctions characterized by immunosuppression of both innate and adaptive immune pathways^46–50^ that likely contributes to the different antibody responses to both antigens. Therefore, this could lead to negative results in diagnosis or response to vaccination aimed to determine or elicit the classes and subclasses of antibodies in this patient population.

Our data revealed distinct classes/subclasses of antibodies against Hcp1 and OPS antigens and some association with some characteristics of melioidosis patients. Based on these findings, diverse classes/subclasses of antibodies may play important roles in humoral immune protection in melioidosis and inform the development of serodiagnostic tests and vaccines.

## Materials and Methods

### Plasma samples

Plasma samples used in this study included 102 samples from culture-confirmed melioidosis patients who were admitted to Udon Thani hospital, Udon Thani, northeast Thailand between July 2015 and September 2017 and 50 serum samples obtained from healthy donors who resided in northeast Thailand. The samples of melioidosis patients were collected at day 0, day 5, day 12 and day 28 after enrollment. Day 0 was the day that *B. pseudomallei* was reported by the microbiology laboratory in any clinical specimen from the patients. The melioidosis patients were grouped for comparison as follows: 54 survivors to 28 days, 48 non-survivors to 28 days, 66 diabetes patients (DM), 36 non-DM patients, 91 patients with bacteremia, 11 patients with non-bacteremia, 14 renal disease patients and 88 non-renal disease patients. Diabetes was characterized by the diabetes clinic at Udon Thani Hospital, Udon Thani, northeast Thailand. Criteria for diabetes diagnosis are as follows: plasma glucose level >200 mg/dl; 75 g oral glucose tolerance test (OGTT) ≥200 mg/dl; hemoglobin A1c (HbA1c) ≥6.5%. Criteria for renal disease are as on the KDIGO 2012 Clinical Practice Guideline for the Evaluation and Management of Chronic Kidney Disease^51^.

### Ethical approval

The study was approved by the Ethics Committee of the Faculty of Tropical Medicine, Mahidol University (approval number MUTM 2015-002-01 and MUTM 2019-035-01) and Udon Thani Hospital, Udon Thani (approval number 0032.102/034), Thailand. Written informed consent was obtained from all subjects enrolled in this study. All research was performed in accordance with relevant guidelines and regulations.

### Preparation of antigens

Recombinant Hcp1 protein (rHcp1) was prepared as previously described^14,19^. The protein concentration was determined using a bicinchroninic acid (BCA) protein assay kit (Pierce, Massachusetts, USA). High Capacity Endotoxin Removal Resin (Pierce) was used for endotoxin removal. A Limulus Amebocyte Lysate (LAL) Chromogenic Endotoxin Quantitation Kit (Pierce) was used for rHcp1 endotoxin quantitation^14^.

OPS was extracted from *B. pseudomallei* strain RR2808 (expresses type A OPS) using a modified hot phenol method as previously described^52,53^. The OPS was purified by acid hydrolysis and gel permeation chromatography as described in previous studies^53,54^.

### ELISA

Antibody classes and subclasses were determined in plasma samples in duplicate using ELISAs based on a rHcp1 (Hcp1-ELISA) and purified OPS (OPS-ELISA) as previously described^14^. The dilution of serum samples and horseradish peroxidase-conjugated secondary antibodies were optimized prior to use. Antibody classes included IgA, IgM and IgG and IgG subclasses included IgG1, IgG2, IgG3 and IgG4.

Briefly, 50 µl of antigen at concentrations of 2.5 µg/ml for Hcp1 and 1.0 µg/ml OPS were coated on each well of 96 well plate (nunc^TM^, Sigma-Aldrich, Darmstadt, Germany) and incubated at 4 °C for overnight. The plate was washed with 300 µl of PBS containing 0.05% tween-20 for 4 times using HydroFlex^TM^ microplate washer (TECAN, Männedorf, Switzerland) and blocked with 200 µl of 5% skim milk in PBS at 37°C for 2 h. Following washing as above, 50 µl plasma samples (diluted in 1% bovine serum albumin, 0.05% Tween-20 in PBS) were added and the plate was incubated at RT for 30 min. After washing, the plate was incubated with 50 µl of optimized dilution of HRP-conjugated anti-human immunoglobulins at RT for 30 min. Dilution of secondary antibodies with HRP conjugates were used as follows: 1:5,000 for HRP-conjugated goat anti-human IgA (Invitrogen, MD, USA); 1:250 for HRP-conjugated rabbit anti-human IgM (Dako, Glostrup, Denmark); 1:6,000 for HRP-conjugated rabbit anti-human IgG (Dako); 1:125 HRP-conjugated mouse anti-human IgG1 (Invitrogen); 1:125 HRP-conjugated mouse anti-human IgG2 (Invitrogen), 1:125 for HRP-conjugated mouse anti-human IgG3 (Invitrogen), and 1:125 for HRP-conjugated mouse anti-human IgG4 (Invitrogen). The plate was washed again and the color was developed by adding 50 µl of 3,3′,5,5′ tetramethylbenzidine (TMB) with peroxidase (Novex, Lifetechnologies, MD, USA). The reaction was stopped after incubating at RT for 15 min by adding 50 µl of 1 N HCl. The absorbance was measured at an optical density (OD) of 450 nm using a Sunrise^TM^ microplate reader (Tecan, Männedorf, Switzerland).

Positive control was pooled culture-confirmed melioidosis patients’ sera (N = 10). Negative control was pooled healthy donors’ sera (N = 10)^13^. The OD value of a blank which contained only an assay diluent was subtracted from all OD values of test samples.

### Statistical analysis

Statistical analysis was performed using GraphPad Prism version 7.0 (GraphPad Software Inc, La Jolla, CA). Antibodies for different host variables were presented as dot plots. The plots present medians with 25^th^ percentile and 75^th^ percentile. Mann-Whitney test was used for testing the difference of medians. Spearman’s rank correlation was used to determine pairwise correlation coefficient (rho) between each antibody pair. Correlations were defined as very high correlation (0.90 to 1.00, −0.90 to −1.00), high correlation (0.70 to 0.90, −0.70- to −0.90), moderate correlation (0.50 to 0.70, −0.50 to −0.70), low correlation (0.30 to 0.50, −0.30 to −0.50), and negligible correlation (0.00 to 0.30, 0.00 to −0.30)^26^. The diagnostic potential of antibody classes and subclasses against Hcp1 and OPS were determined by plotting receiver operating characteristics (ROC). The area under ROC curve (AUROCC) of each antibody response was calculated for increasing percentages of true-positive rate (% sensitivity) and false-positive rate (1- % specificity). The association between antibody response and continuous variables (age and days of clinical symptoms) were performed using linear regression analysis. The association of antibody responses and host factors (DM, bacteremia, gender, renal disease and mortality) was performed using logistic regression analysis.

## Supplementary information

Supplementary information
