# Supplementary material for: Distinct classes and subclasses of antibodies to hemolysin co-regulated protein 1 and O-polysaccharide and correlation with clinical characteristics of melioidosis patients
Source: Sci Rep. 2019 Sep 27;9:13972. doi: 10.1038/s41598-019-48828-4 (PMC6764960; doi:10.1038/s41598-019-48828-4)

Distinct classes and subclasses of antibodies to hemolysin co-regulated protein 1 and O-polysaccharide and correlation with clinical characteristics of melioidosis patients

Apinya Pumpuang[1,2], Rungnapa Phunpang[3], Peeraya Ekchariyawat[4], Adul Dulsuk[3], Siriorn Loupha[3], Kochnipa Kwawong[3], Yaowaree Charoensawat[1], Ekkachai Thiansukhon[5], Nicholas P.J. Day[3,6], Mary N. Burtnick[7], Paul J. Brett[7], T. Eoin West[8], Narisara Chantratita[1,3*]

[1]Department of Microbiology and Immunology, Faculty of Tropical Medicine, Mahidol University, Bangkok, Thailand

[2]Department of Clinical Pathology, Faculty of Medicine, Vajira Hospital, Navamindradhiraj University, Bangkok, Thailand

[3]Mahidol-Oxford Tropical Medicine Research Unit, Faculty of Tropical Medicine, Mahidol University, Bangkok, Thailand

[4]Department of Microbiology, Faculty of Public health, Mahidol University, Bangkok, Thailand

[5]Department of Medicine, Udon Thani Hospital, Udon Thani, Thailand

[6]Center for Tropical Medicine and Global Health, University of Oxford, Oxford, United Kingdom

[7]Department of Microbiology and Immunology, University of Nevada, Reno School of Medicine, Reno, Nevada, USA

[8]Division of Pulmonary and Critical Care Medicine, Harborview Medical Center, and International Respiratory and Severe Illness Center, University of Washington, Seattle, WA, USA

*Corresponding author

E-mail: narisara@tropmedres.ac; narisara.cha@mahidol.ac.th

Supplementary information

Supplementary Figure S1 Correlation (rho) of different classes and IgG subclasses of antibody responses to Hcp1 (a), OPS (b), and correlation of the same classes and IgG subclasses of antibodies against Hcp1 and against OPS (c) in plasma samples of melioidosis patients. The correlation of antibodies was analysed at serum dilution 1:250.

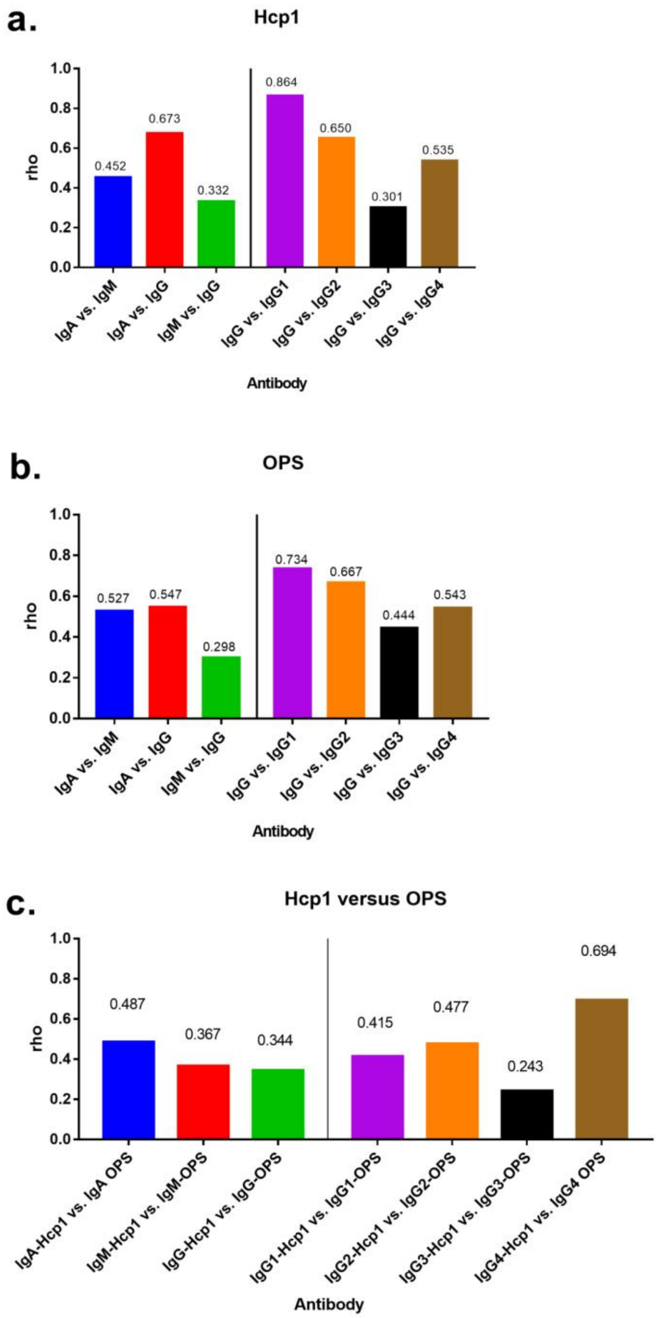

Supplementary Figure S2 Scatter plots of age and different classes and IgG subclasses of antibody responses to Hcp1 (a), OPS (b)

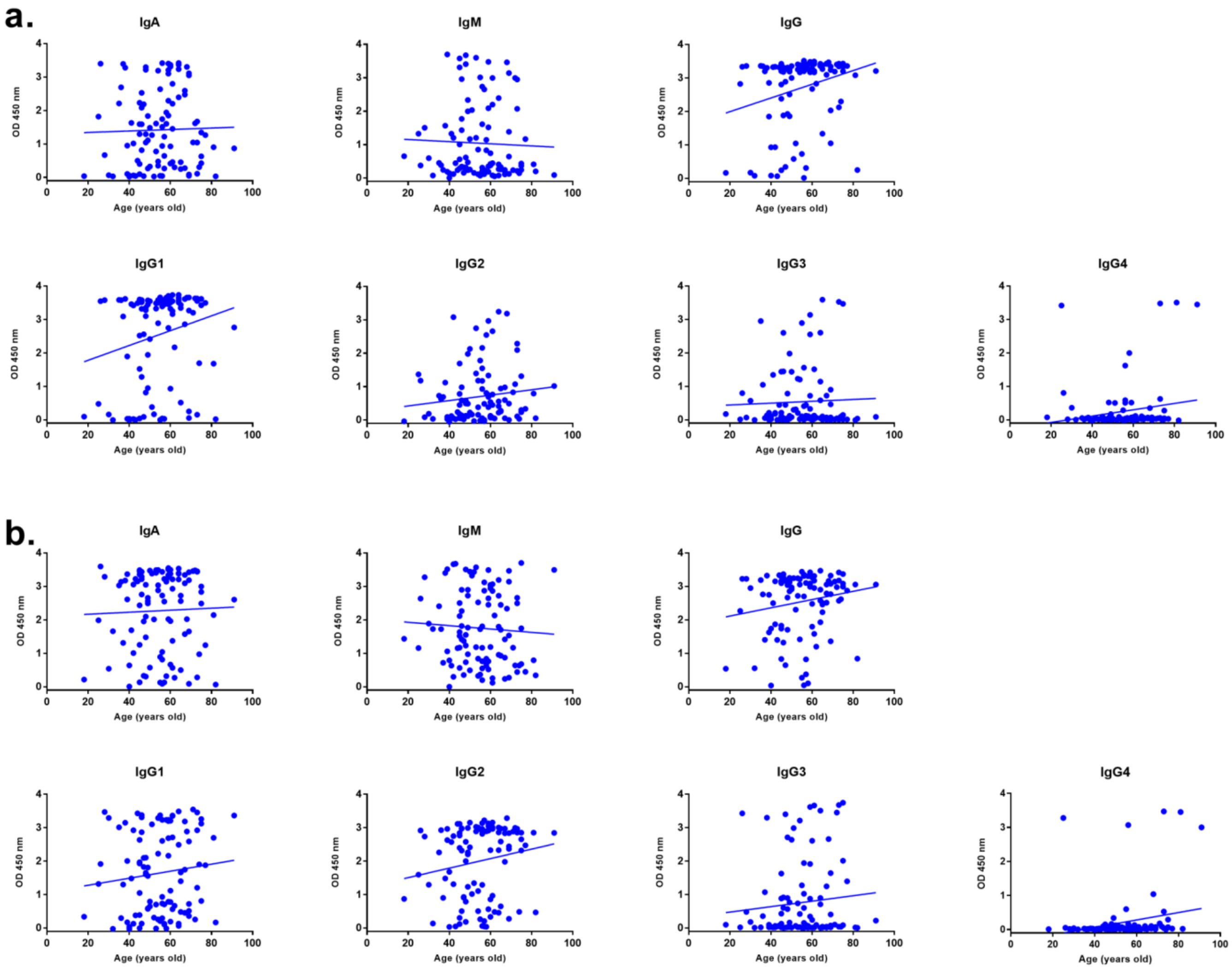

Supplementary Figure S3 Longitudinal analysis of classes and subclasses of antibody against Hcp1 in survivors melioidosis patients and antibody responses at day 0 in non-survivors melioidosis patients. ELISAs were performed in the plates coated with Hcp1 antigens using serum samples from melioidosis patients at dilution of 1:250. Scatter plots represent antibody levels. Median line and 25th and 75th percentile boundaries are shown.

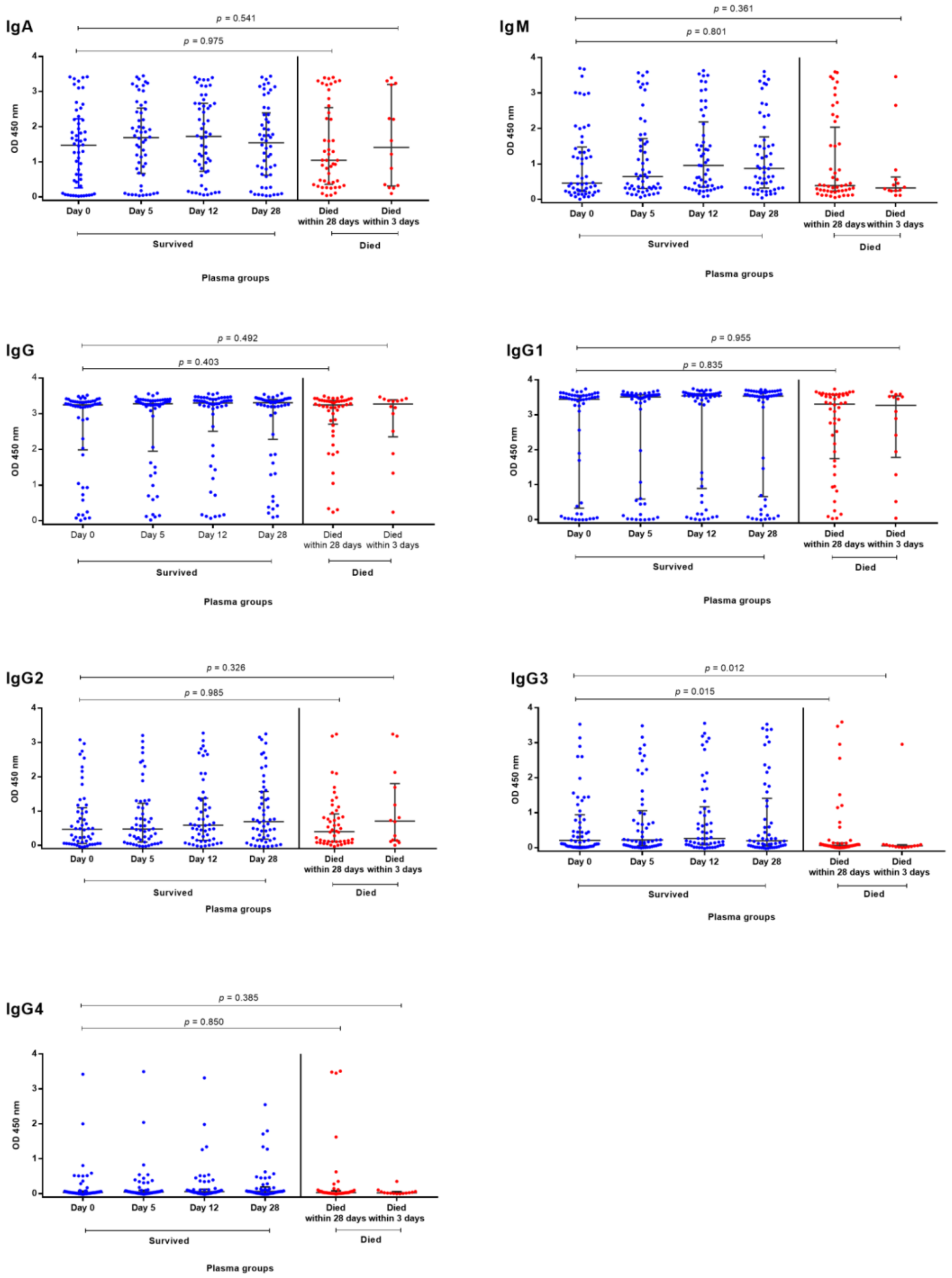

Supplementary Figure S4 Longitudinal analysis of classes and subclasses of antibody against OPS in survivors melioidosis patients and antibody responses at day 0 in non-survivors melioidosis patients. Antibodies were determined by ELISA using plasma samples from melioidosis patients at dilution of 1:250. Scatter plots represent antibody levels of individual patients. Median line and 25th and 75th percentile boundaries are shown.

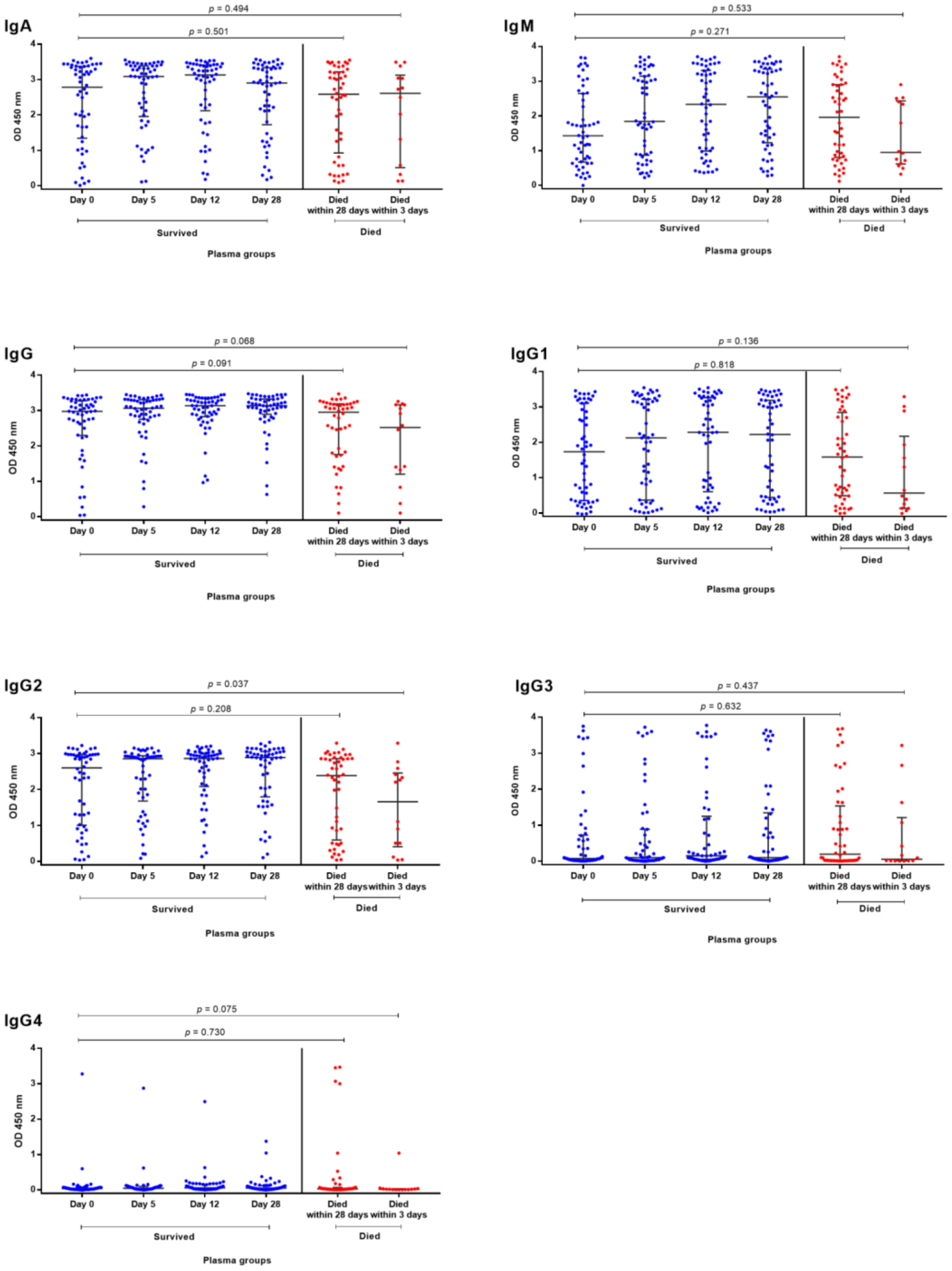

Supplementary Figure S5 Effect of bacteremia on classes and subclasses of antibodies response against Hcp1 (a) and OPS (b) in plasma samples of melioidosis patients. The plasma samples were diluted at serum dilution 1:250 for ELISAs.

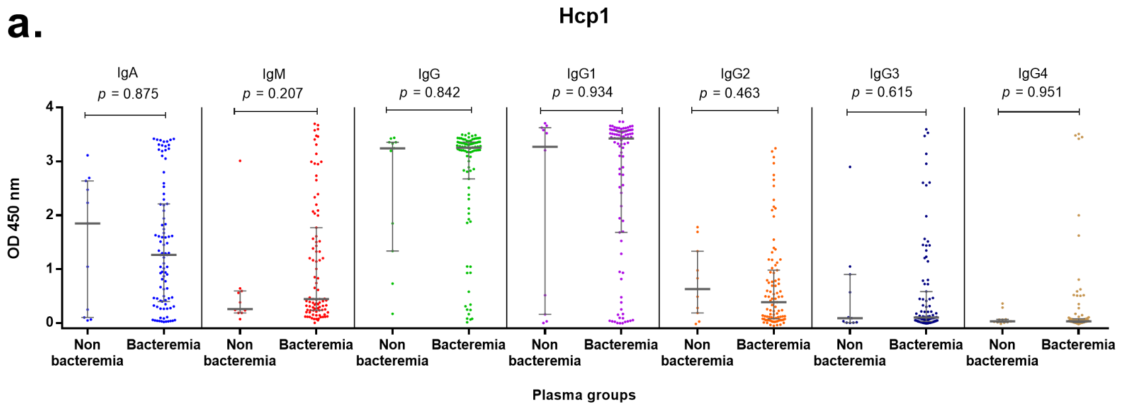

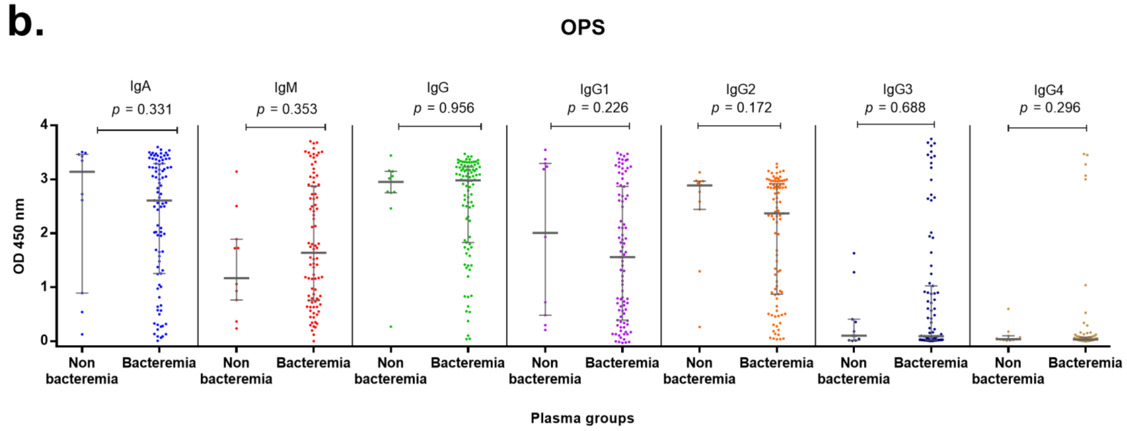

Supplementary Figure S6 Heat map of longitudinal analysis of classes and subclasses of antibody responses to Hcp1 and OPS in plasma samples of melioidosis patients with bacteremia and non-bacteremia melioidosis. The antibody levels were determined at day 0, day 5, day 12 and day 28 by ELISAs using plasma dilution 1:250.

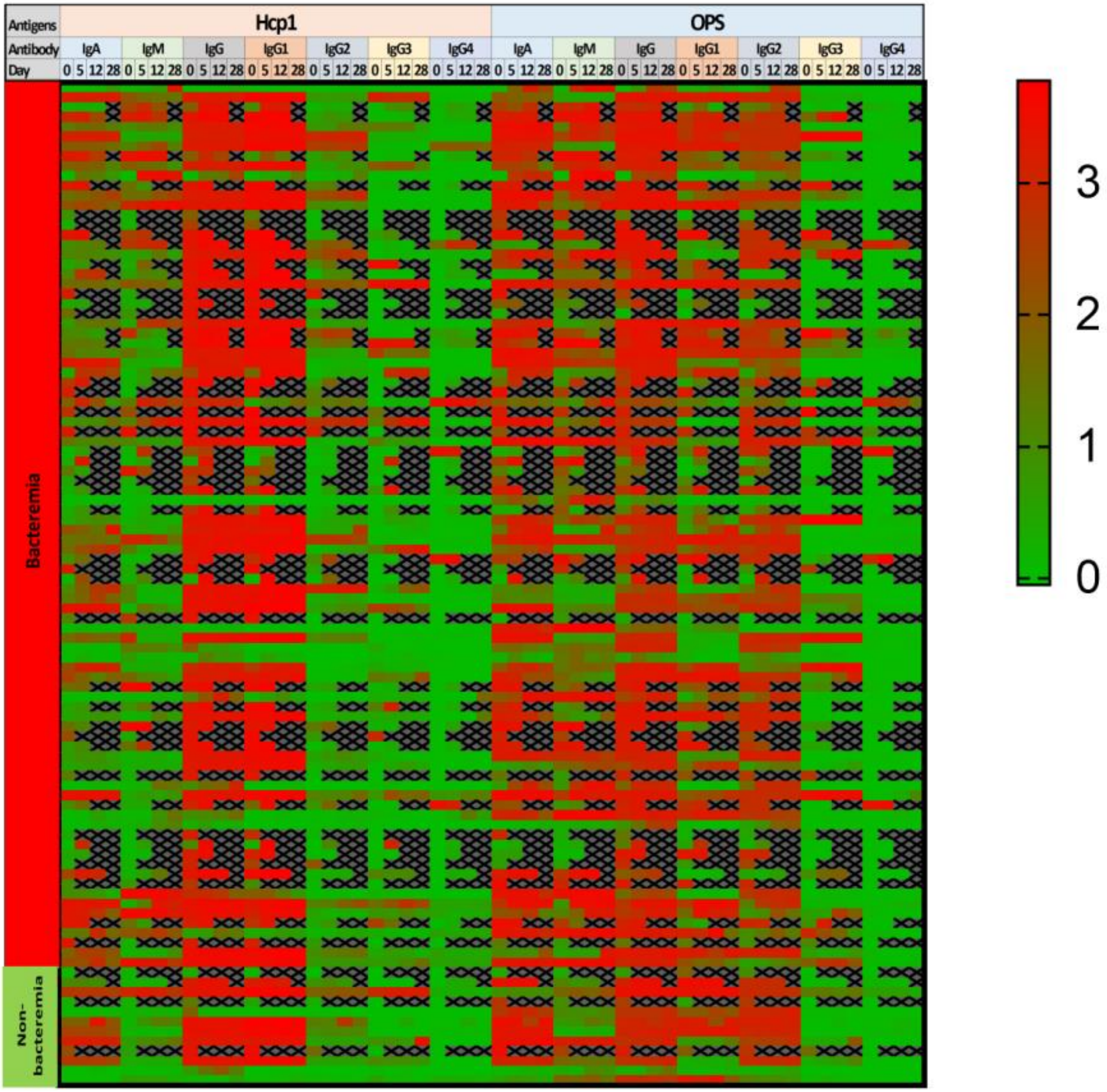

Supplementary Figure S7 Effect of gender on classes and subclasses of antibodies response against Hcp1 (a) and OPS (b) in plasma samples of melioidosis patients. The plasma samples were diluted at serum dilution 1:250 for ELISAs.

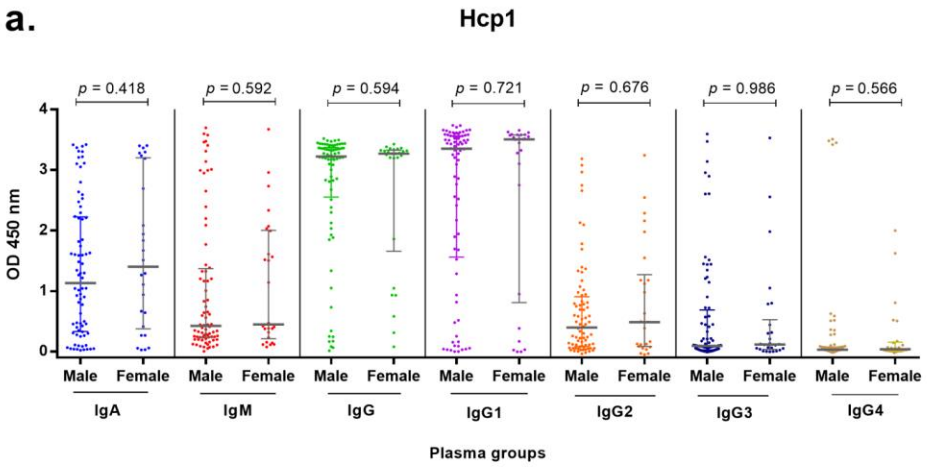

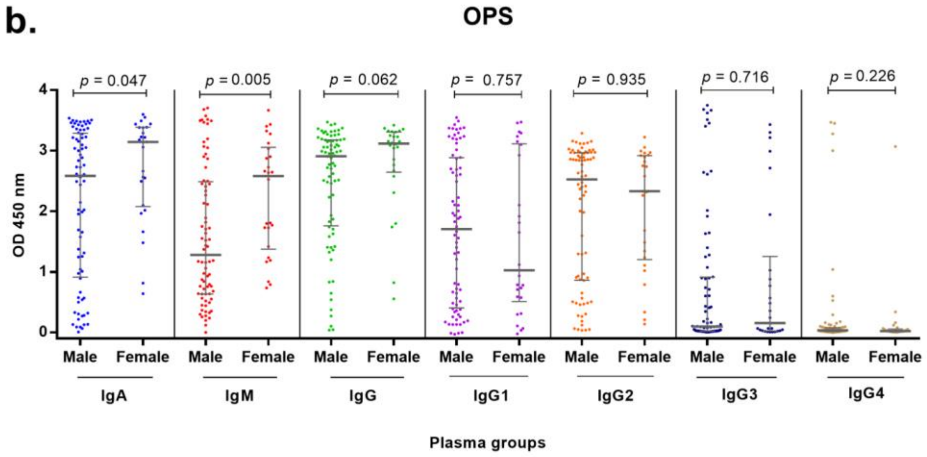

Supplementary Figure S8 Effect of diabetes on classes and subclasses of antibodies response against Hcp1 (a) and OPS (b) in plasma samples of melioidosis patients. The plasma samples were diluted at serum dilution 1:250 for ELISAs.

a.

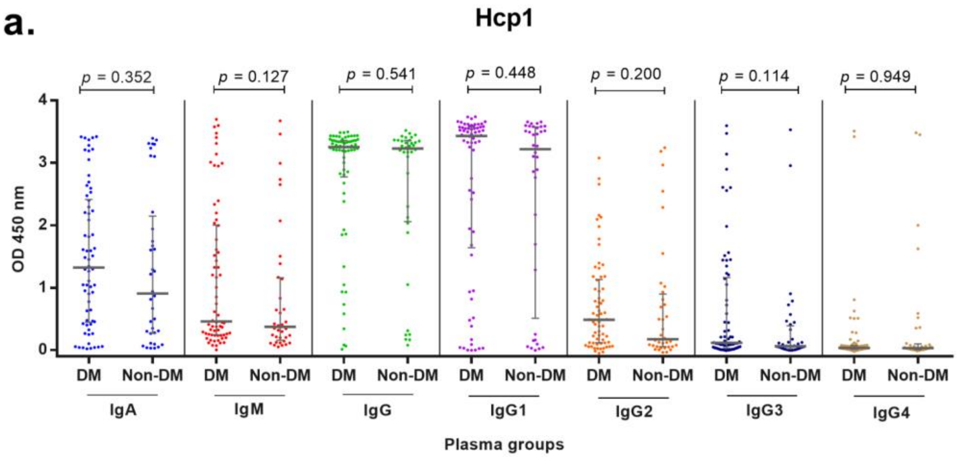

b.

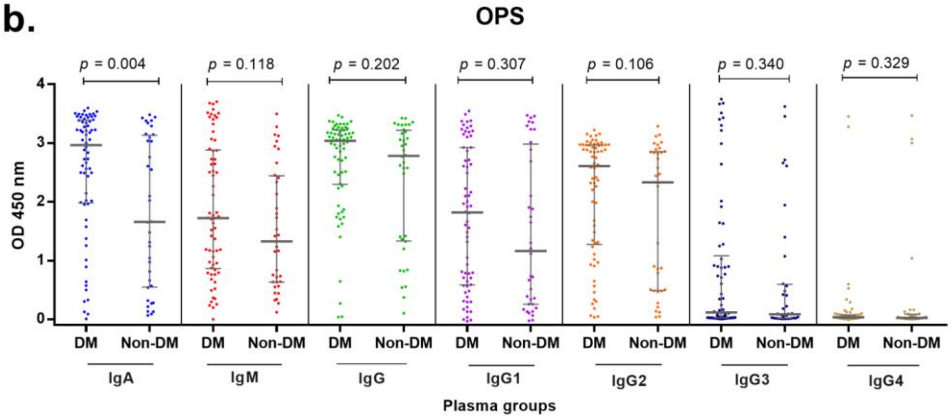

Supplement: Supplementary file 1 — Supplementary information [file 41598_2019_48828_MOESM1_ESM.pdf]
